# Hybrid Modeling of the Reversed‐Phase Chromatographic Purification of an Oligonucleotide: Few‐Shot Learning From Differentiable Physics Solver‐in‐the‐Loop

**DOI:** 10.1002/bit.29018

**Published:** 2025-05-09

**Authors:** Yu‐Cheng Chen, Ismaele Fioretti, Dong‐Qiang Lin, Mattia Sponchioni

**Affiliations:** ^1^ Key Laboratory of Biomass Chemical Engineering of Ministry of Education, Zhejiang Key Laboratory of Smart Biomaterials, College of Chemical and Biological Engineering Zhejiang University Hangzhou China; ^2^ Department of Chemistry, Materials and Chemical Engineering “Giulio Natta” Politecnico di Milano Milano Italy

**Keywords:** differentiable physics, few‐shot learning, hybrid model, oligonucleotides, reversed‐phased chromatography

## Abstract

Hybrid models integrate mechanistic and data‐driven components, effectively addressing the challenges of limited process understanding and data availability typical of biopharmaceutical processes. In this study, we applied a hybrid modeling framework named differentiable physics solver‐in‐the‐loop (DP‐SOL) to describe the reversed‐phase chromatographic purification of an oligonucleotide, overcoming the mentioned limitations of purely mechanistic and data‐driven models. The framework establishes a connection between neural networks (NNs) and mechanistic models through differentiable physical operators and their gradients. We first collected a data set comprising six linear gradient elution experiments at different resin loadings and gradient slopes, split in three experiments each for training and testing, for few‐shot learning. The hyperparameters were determined through a grid search, resulting in a NN with two hidden layers and 14 nodes. Compared to a calibrated mechanistic model used for initialization of NN, the DP‐SOL hybrid model showed significant performance improvement on both training and testing sets, with $R^{2}\;>$ 0.97 for the former. The good predictivity of DP‐SOL is attributed to the combination of mechanistic models and NNs at the solver level. As a novel and versatile hybrid modeling paradigm, DP‐SOL has the potential to significantly impact modeling approaches in the downstream processing field and the broader biopharmaceutical sector.

## Graphical abstract

The DP‐SOL hybrid modeling framework was employed to model the reversed‐phase chromatographic purification of oligonucleotides. By combining differentiable physics and neural networks at the solver level, DP‐SOL outperforms traditional mechanistic models in prediction accuracy, captures complex process dynamics and offers a robust approach for downstream biopharmaceutical process modeling with limited data availability.

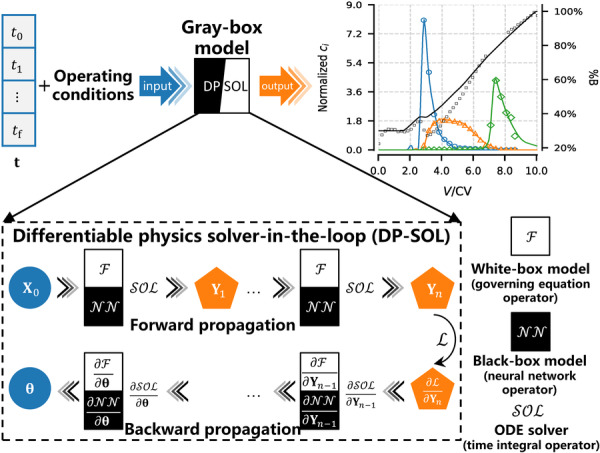

## 1 Introduction

Mathematical models of chemical/biochemical processes can be divided into two main classes (Rizki and Ottens 2023): mechanistic models, expressing fundamental principles on the process in the form of partial differential equations (PDEs) (Chen et al. 2024), and data‐driven models predominantly based on deep learning (DL). While mechanistic models can provide a deeper insight into the process (Huuk et al. 2014; Kozorog et al. 2023; Qian et al. 2023), they require a comprehensive understanding of the system under investigation, which is often out of reach. In contrast, data‐driven models require large data sets to establish statistical correlations between input and output variables (Velioğlu et al. 2024) and may lack interpretability and process insights.

Hybrid models, often referred to as gray‐box models, have emerged as a valuable alternative as they combine the strengths of purely mechanistic and purely data‐driven models, compensating the limits of both (Jungbauer et al. 2024; Mahanty 2023; Malinov et al. 2024; Narayanan et al. 2022; Narayanan et al. 2023; Roush et al. 2020; Wittkopp et al. 2024). This integration, defined as physics‐based DL by Thuerey et al. (2021), encompasses three categories: supervised learning, physics‐informed neural networks (PINNs), and differentiable numerical simulations of physical systems (referred to as differentiable physics (DP) in this study). Thuerey et al. (2021) suggested that these methods are evolutionary, with DP representing the ultimate form of physics‐based DL.

Recent attempts at supervised learning (first‐category of physics‐based DL) in the biopharmaceutical field include (Ding et al. 2023; Narayanan et al. 2022; Narayanan et al. 2022; Narayanan et al. 2021). In cell culture processes (upstream), these hybrid models have shown superior performance compared to purely mechanistic and purely data‐driven models (Narayanan et al. 2022; Narayanan et al. 2022). In downstream processing, neural networks (NNs) were used to describe the adsorption dynamics, which are poorly understood, while retaining a mechanistic description of the molecule transport in the column, thus obtaining effective hybrid NN‐PDE models (Ding et al. 2023; Narayanan et al. 2021). However, this supervised learning has two major limitations. First, its performance heavily depends on the integration of the first‐principles and data‐driven component, which lacks a unified approach and requires extensive trial and error. For example, in chromatographic modeling (Ding et al. 2023; Narayanan et al. 2021), the different mass transfer contributions can be modeled using either NNs or PDEs, and the integration of these terms needs careful consideration. Second, this model was trained using least squares optimizations and disregards the differentiability and backpropagation of NNs, making it effective only with shallow NNs. When NNs are deepened to model more complex processes, this method becomes inapplicable, easily falling into local optima.

The second category, PINNs, has also been reported in chromatography modeling for both forward and inverse problems (Santana et al. 2022; Söderström 2022; Subraveti et al. 2022a, 2022b, 2023; Tang et al. 2023; Zou et al. 2024). However, the primary issue with PINNs is that they build independently of traditional numerical methods, discarding decades of accumulated knowledge in numerical computation.

To overcome these limitations, a novel and versatile hybrid modeling framework, DP (third‐category physics‐based DL), was proposed. Unlike supervised learning, which introduces NNs at the model level, DP integrates NNs at the solver level (Ramsundar et al. 2021). This solver‐level integration allows existing numerical solvers to compute gradients with respect to their inputs, leading to a DP solver. Once this integration is achieved for all computations within a simulation, the automatic differentiation (AD) functionality of DL frameworks combined with backpropagation enables gradient‐based model training, which significantly outperforms the least squares optimization methods (first category physics‐based DL). Therefore, DP overcomes the limitations of the first category of physics‐based DL by eliminating the need for a careful integration of NN and PDE and by leveraging the differentiability and backpropagation of NNs. Additionally, it addresses the shortcomings of the second category of physics‐based DL by retaining numerical computation knowledge.

A classical approach involves the cyclic evaluation of DP using differentiability and backpropagation within the solver time‐stepping process, termed as DP solver‐in‐the‐loop (DP‐SOL), which can significantly reduce numerical simulation errors. Since its introduction in 2020, DP‐SOL has been employed to develop various hybrid models in computational fluid dynamics, covering the Navier‐Stokes equations (Um et al. 2020), and convection‐dispersion problems (Wiewel et al. 2020). Given its potential, chromatographic models, a variant of convection‐dispersion systems, can greatly benefit in terms of accuracy and computational time by the implementation of DP‐SOL. In particular, reversed‐phase chromatography (RPC) is one of the most frequently used processes for the purification of biopharmaceuticals (Catani et al. 2020; De Luca et al. 2020), but it is still predominantly described through mechanistic models, including the Mollerup (2007) model based on thermodynamic properties and activity (Chen et al. 2024), as well as stoichiometric displacement models (SDM) (Arkell et al. 2017; Arkell et al. 2018; Arkell et al. 2018) with their simplified version (Nozaki et al. 2024). However, the lack of consensus on the underlying mechanisms of RPC, particularly concerning the use of activity models for organic solvents, has limited the applications of these mechanistic approaches in this field.

Given the limited understanding of RPC processes, hybrid models represent a promising alternative for a reliable description of the operation. However, hybrid models require more experimental data for training compared to purely mechanistic models, due to the incorporation of data‐driven components. At the same time, the generation of a sufficient amount of high‐quality data for model training remains a challenge in biotechnology (Jungbauer et al. 2024; Ou et al. 2024; Saleh et al. 2022; Wu et al. 2024). Consequently, this field often resorts to few‐shot learning (Liu et al. 2024) to reduce the data requirement and align with mechanistic models, thus facilitating the transition from a more conventional approach to the hybrid one without incurring into additional experimental effort.

In this direction, this study aims at developing a hybrid model using DP‐SOL that can accurately describe the RPC purification of an oligonucleotide with limited experimental data. First, we introduced the concept and derivation of DP‐SOL and discussed how to construct it for RPC processes. Then, DP‐SOL was implemented in the modeling of six fractionated linear gradient separations obtained at different oligonucleotide loadings on the resin and gradient durations. Out of these experiments, three were used for training the model and three for testing it, in a 1:1 ratio. A comparison between the DP‐SOL and a mechanistic model was conducted using the same data set, showing the great potential of the hybrid model in improving the accuracy and predictivity of the simulations and paving the way to a new paradigm in chromatography modeling.

## 2 Theory

**Figure 1:**
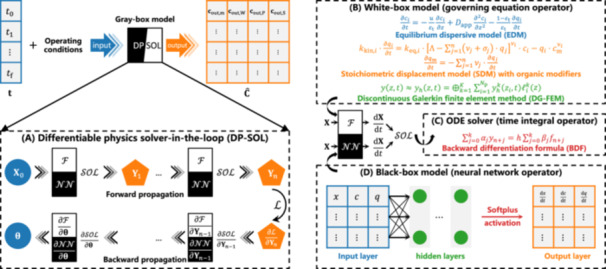
Differentiable physics solver‐in‐the‐loop (DP‐SOL) for hybrid modeling (gray‐box model). (A) Forward and backward propagation of DP‐SOL with solver‐in‐the‐loop using differentiable operators F, NN, and SOL. (B) White‐box model for operator F. (C) Solver for operator SOL. (D) Black‐box model for operator NN.

Figure 1 schematically illustrates the concept of DP‐SOL for the hybrid modeling of RPC processes and the different panels are described in details in the following sections.

### 2.1 Differentiability and Backpropagation of Neural Networks

As shown in Figure 1D, DP‐SOL is implemented by a fully connected NN (FCNN) comprising one input layer, $n_{L}$ hidden layers, and one output layer, with a forward propagation scheme:

(1) $$Y^{l}=W^{l}X^{l-1}+b^{l},$$

(2) $$X^{l}=f^{l}\left(Y^{l}\right),$$

where $Y^{l}$, $X^{l}$, $W^{l}$, $b^{l}$, and $f^{l}$ denote the output, input, weights, bias, and activation function of the l‐th layer, respectively.

The training of NNs can be summarized as the tuning of the parameters θ to minimize the loss function L, given the input and output, so that the NN output can approximate the given output as closely as possible. This can be expressed as the following optimization problem:

(3) $$\min_{\theta }L,$$

(4) $$\theta =\left[W^{0}W^{1}\cdots W^{n_{L}}b^{0}b^{1}\cdots b^{n_{L}}\right].$$

The differentiability and backpropagation of NNs allow for updating θ by gradient descent methods:

(5) $$W^{l}=W^{l}-\alpha \frac{\partial L}{\partial W^{l}},$$

(6) $$b^{l}=b^{l}-\alpha \frac{\partial L}{\partial b^{l}},$$

where α denotes the learning rate, while the gradients $\frac{\partial L}{\partial W^{l}}$ and $\frac{\partial L}{\partial b^{l}}$ can be obtained by:

(7) $$\frac{\partial L}{\partial W^{l}}={\left(X^{l-1}\right)}^{T}\times \frac{\partial L}{\partial Y^{l}},$$

(8) $$\frac{\partial L}{\partial b^{l}}=1\times \frac{\partial L}{\partial Y^{l}},$$

(9) $$\frac{\partial L}{\partial X^{l-1}}=\frac{\partial L}{\partial Y^{l}}\times {\left(W^{l}\right)}^{T}.$$

### 2.2 Solver for Addressing the Initial Value Problem in Chromatography

The unknowns y determined by chromatographic models include the concentrations of each specie in the mobile phase (c) and in the stationary phase (q) at different times (t) and longitudinal position (z):

(10) $$y\left(z,t\right)={\left[c\left(z,t\right)\;q\left(z,t\right)\right]}^{T}.$$

The governing equation of Equation (10) can be written as:

(11) $$\frac{\partial y}{\partial t}\left(z,t\right)={\left[\frac{\partial c}{\partial t}\left(z,t\right)\;\frac{\partial q}{\partial t}\left(z,t\right)\right]}^{T},$$

whose right‐hand side can be reduced to a spatially semi‐discretized form, resulting in the approximate solution $y_{h}$, rewritten as:

(12) $$\frac{dy_{h}\left(t\right)}{dt}=f\left(y_{h}\left(t\right),t\right).$$

Given the initial value $y_{h}\left(0\right)=y_{0}$ and the residual term f, one can integrate over time using a solver to obtain $y_{h}$ for any given time t. Equation (12) can be efficiently solved by the backward differentiable formula (BDF), which uses high‐order accuracy whenever possible, resorting to lower order when necessary to maintain stability, with the order ranging from one to five. The sth‐order BDF method (Figure 1C) can be expressed as:

(13) $$\alpha_{0}y_{n}+\alpha_{1}y_{n+1}+\cdots +\alpha_{s}y_{n+s,}=h\left(\beta_{0}f_{n}+\beta_{1}f_{n+1}+\cdots +\beta_{s}f_{n+s}\right),$$

where α and β are the coefficients of the BDF method, and h is the time step. $y_{n+j}$ and $f_{n+j}$ represent $y_{h}\left(t_{n+j}\right)$ and $f\left(y_{n+j},t_{n+j}\right)$, respectively. The BDF method requires iteration to determine the solution at the following time step. If Equation (13) is expressed as a linear system, each iteration involves solving Equation (14)

(14) Ay=f,

where $y={\left[y_{n}\;y_{n+1}\;\cdots \;y_{n+s}\right]}^{T}$, the coefficient matrix A depends on s and α, and the right‐hand vector f depends on $h\sum_{j=0}^{s}\beta_{j}f_{n+j}$.

### 2.3 Solver With Differentiable Operators

This section shows how to integrate the solver with the differentiability and backpropagation of NNs to construct DP‐SOL for hybrid modeling. The temporal integration in Equation (13) approximates the unknown $y_{n+1}$ using the known $y_{n}$, which can be understood as applying a series of operators $P_{1}$, $P_{2}$, …, $P_{m}$ to $y_{n}$ such that

(15) $$P_{1}\circ P_{2}\circ \cdots \circ P_{m}\circ y_{n}=y_{n+1},$$

where ∘ represents function decomposition, i.e., $P_{m}\circ y_{h}\left(t\right)=P_{m}\left(y_{h}\left(t\right)\right)$. These operators can be some form of transformation or operation. For instance, a matrix is considered a linear operator that maps one vector space to another. The BDF temporal integration in Equation (14) is an operator, which maps y to f by operator A. Similarly, the forward propagation of NNs in Equation (1), is also an operator that maps $X^{l-1}$ to $Y^{l}$ by operators $W^{l}$ and $b^{l}$. Therefore, if we combine the temporal integration and the forward propagation into one solver, all computation processes in this solver can be expressed in the form of operators.

We refer to these processes as the forward propagation of DP‐SOL (Figure 1A), which is expressed by the operator F for the spatial discretization of the governing equation according to Equtaion (12), by the operator SOL for the temporal integration of the BDF solver through Equation (14), and by the operator NN for the forward propagation of NNs in Equations (1) and (2):

(16) $$Y_{n}=SOL\left[F\left(X_{n-1}\right)\cdot NN\left(X_{n-1}\right)\right],$$

(17) $$X_{n}=Y_{n}.$$

Similarly, to implement the backpropagation of DP‐SOL (Figure 1A), it is necessary that both NNs and the BDF solver can perform backpropagation. The NN backpropagation is based on its differentiability. So, the BDF solver also needs differentiability. Since the BDF solver can be expressed by differentiable operators, it is differentiable by taking the partial derivative of both sides of Equation (15) with respect to $y_{n}$:

(18) $$\frac{\partial \left(P_{1}\circ P_{2}\circ \cdots \circ P_{m}\circ y_{n}\right)}{\partial y_{n}}=\frac{\partial y_{n+1}}{\partial y_{n}},$$

which is rewritten by the definition of differentiable operators and the chain rule as:

(19) $$\frac{\partial P_{m}\left(y_{n}\right)}{\partial y_{n}}\frac{\partial P_{m-1}\left(P_{m}\right)}{\partial y_{n}}\cdots \frac{\partial P_{1}\left(P_{2}\right)}{\partial y_{n}}=\frac{\partial y_{n+1}}{\partial y_{n}},$$

where $\partial P_{m}/\partial y_{n}$ represents the Jacobian matrix. Thus, $\partial y_{n+1}/\partial y_{n}$ can be obtained.

Combining the derivations of forward and backward propagation reported above, we obtain DP‐SOL as shown in Figure 1A, where all calculations can be represented in terms of differentiable operators. DP‐SOL is iteratively evaluated during time stepping. When the contribution of NN in Equation (16) is equal to one, DP‐SOL degenerates into a general numerical method. Therefore, the contribution of NN can be understood as a correction to the output of governing equation F or the input of SOL. DP‐SOL has strong generalization abilities and can be applied to model various processes by altering the governing equations, discretization forms, solvers, and NN structures.

### 2.4 Mechanistic Models and Numerical Solution

DP‐SOL involves three operators. SOL (Figure 1C) and NN (Figure 1D) have been identified above, leaving only F (Figure 1B), which comprises contributions from both the governing equation and its discrete form. In this study, F is adapted to the description of RPC processes. The equilibrium‐dispersive and dispersed plug flow models are used for the adsorbate components i and the modifier m (organic solvents), respectively:

(20) $$\frac{\partial c_{i}}{\partial t}\left(z,t\right)=-\frac{u}{\varepsilon_{t}}\frac{\partial c_{i}}{\partial z}\left(z,t\right)+D_{\text{app}}\frac{\partial^{2}c_{i}}{\partial z^{2}}\left(z,t\right)-\frac{1-\varepsilon_{t}}{\varepsilon_{t}}\frac{\partial q_{i}}{\partial t}\left(z,t\right),$$

(21) $$\frac{\partial c_{m}}{\partial t}\left(z,t\right)=-\frac{u}{\varepsilon_{t}}\frac{\partial c_{m}}{\partial z}\left(z,t\right)+D_{\text{app}}\frac{\partial^{2}c_{m}}{\partial z^{2}}\left(z,t\right),$$

where u, $\varepsilon_{t}$, and $D_{\text{app}}$ are the superficial velocity, total porosity, and apparent axial dispersion coefficient, respectively. A simplified SDM is employed for the stationary phase (Arkell et al. 2017; Mollerup 2007):

(22) $$k_{\text{kin},i}\cdot \frac{\partial q_{i}}{\partial t}\left(z,t\right)=k_{\text{eq},i}\cdot {\left[\Lambda -\sum_{j=1}^{n}\left(\nu_{j}+\sigma_{j}\right)\cdot q_{j}\left(z,t\right)\right]}^{\nu_{i}}\cdot c_{i}\left(z,t\right)-q_{i}\left(z,t\right)\cdot c_{m}^{\nu_{i}}\left(z,t\right),$$

(23) $$\frac{\partial q_{m}}{\partial t}\left(z,t\right)=-\sum_{j=1}^{n}\nu_{j}\cdot \frac{\partial q_{j}}{\partial t}\left(z,t\right),$$

where Λ represents the ligand density. $k_{\text{kin}}$, $k_{\text{eq}}$, ν, and σ represent the model parameters, namely the kinetic coefficient, equilibrium constant, characteristic charge, and shielding factor. For both adsorbate components i and the modifier m, Danckwerts and Neumann boundary conditions are applied at the inlet (z=0) and outlet (z=L) of the chromatographic column, respectively:

(24) $$-\frac{\varepsilon_{t}D_{\text{app}}}{u}\frac{\partial c}{\partial z}\left(0,t\right)+c\left(0,t\right)=\left\{\begin{array}{ll} c_{\text{inj}} & 0<t\leq t_{\text{inj}}\\ 0 & t>t_{\text{inj}} \end{array}\right.,$$

(25) $$\frac{\partial c}{\partial z}\left(L,t\right)=0,$$

where $c_{\text{inj}}$ and $t_{\text{inj}}$ represent the injection concentration and time, respectively. As initial conditions, we assumed a fully regenerated and equilibrated column.

Another essential element of operator F is the discrete form (obtaining Equation (12) from Equation (11)). A discontinuous Galerkin finite element method (DG‐FEM) with order $n_{p}$, number of elements K, and Legendre polynomial basis functions l is used:

(26) $$y\left(z,t\right)\approx y_{h}\left(z,t\right)=\overset{K}{\underset{k=1}{⨁}}\sum_{i=1}^{n_{p}}y_{h}^{k}\left(z_{i},t\right)l_{i}^{k}\left(z\right).$$

Details of the DG‐FEM method for solving chromatographic models can be found in Breuer et al. (2023); Javeed et al. (2011); Meyer et al. (2020).

### 2.5 Integration of DP‐SOL With RPC Models

DP‐SOL is coupled with the RPC mechanistic models. For a ternary separation system, including a weakly adsorbed impurity (W), a main product (P), and a strongly adsorbed impurity (S), and linear gradient elution performed with an organic modifier, DP‐SOL can be represented as:

(27) $$\hat{C}=\mathrm{DPSOL}\left(t\right),$$

(28) $$t={\left[t_{0}\;t_{1}\;\cdots \;t_{f}\right]}_{1\times n_{t}}^{T},$$

(29) $$\hat{C}={\left[c_{\text{out},m}\;c_{\text{out},W}\;c_{\text{out},P}\;c_{\text{out},S}\right]}_{n_{t}\times 4},$$

where the total inputs are a time series t and other operating conditions. $t_{f}$ is the final time of the simulation, and the series length $n_{t}$ depends on the sampling frequency of the fraction collector. The total output is the mobile‐phase concentration at the column outlet $\hat{C}$, where each element is a vector to t.

In each iteration of DP‐SOL, the input to F is:

(30) $${\left[c_{m}\;c_{W}\;c_{P}\;c_{S}\;q_{m}\;q_{W}\;q_{P}\;q_{S}\right]}_{\left[K\cdot \left(n_{p}+1\right)\right]\times 8},$$

where each element is a vector with respect to z. $K\cdot \left(n_{p}+1\right)$ implies its length is determined by the discrete order and the number of elements. The output of F is:

(31) $${\left[\frac{dc_{m}}{dt}\;\frac{dc_{W}}{dt}\;\frac{dc_{P}}{dt}\;\frac{dc_{S}}{dt}\;\frac{dq_{m}}{dt}\;\frac{dq_{W}}{dt}\;\frac{dq_{P}}{dt}\;\frac{dq_{S}}{dt}\right]}_{\left[K\cdot \left(n_{p}+1\right)\right]\times 8}.$$

The input to NN is obtained by excluding $q_{m}$ in Equation (30), and normalized by:

(32) $${\tilde{c}}_{i}=\frac{1}{c_{\text{inj},i}}\cdot c_{i},$$

(33) $${\tilde{q}}_{i}=\frac{1}{c_{\text{inj},i}}\cdot q_{i},$$

where the normalization of modifier concentration is unnecessary. The output of NN retains only the last three terms of Equation (31), indicating that the correction of DP‐SOL is only for the adsorption isotherm.

The output of SOL is similar to Equation (30), while its input is the product of F output and NN output. The same normalization of Equations (32) and (33) is applied to the total output of DP‐SOL, resulting in the normalized total output $\hat{C}$. This, along with the observed C, constitutes the mean squared error (MSE):

(34) $$L=\frac{\|\hat{C}-C{\|}_{L^{2}}^{2}}{\|C{\|}_{L^{2}}^{2}}.$$

## 3 Materials and Methods

### 3.1 Experiments

The oligonucleotide utilized was a 20‐mer single‐stranded DNA (5'‐ATA CCG ATT AAG CGA AGT TT‐3) provided by YMC Japan. The RPC experiments were conducted on a ContiChrom CUBE30+ (YMC ChromaCon), which included an external BlueShadow 40D UV/Vis detector set at 300 nm and an external Azura CT2.1 column thermostat with solvent pre‐heat cartridge operated at 50°C, both from Knauer. A YMC Triart C18‐S column, 100 mm in length, 4.6 mm in internal diameter, particle size of 10 μm and pore size of 12 nm with a total porosity of 0.54 was used for elution experiments. The column specific parameter Λ was determined via silanol titration experiments (Arkell et al. 2017), $\varepsilon_{t}$ was determined by pulse injection experiments using NaCl as a nonbinding tracer (Chen et al. 2024), while $D_{\text{app}}$ was calculated from column efficiency via u, column length L and stage number N:

(35) $$D_{\text{app}}=\frac{uL}{2\varepsilon_{t}N}.$$

For elution experiments, buffer A consisted of 99% 0.2 M sodium acetate and 1% acetonitrile, while buffer B comprised 90% 0.2 M sodium acetate and 10% acetonitrile. The operating conditions (buffer composition, velocity, and volume) are detailed in Table 1, and loading volumes and gradient lengths are reported in Table 2. The measured conductivity was converted to %B using the Kohlrausch's law (Carta and Jungbauer 2020).

**Table 1:** Operating conditions of linear gradient elution experiments. CV: column volume.

Step	Buffer (%B)	Velocity (cm/h)	Volume (CV)
Equilibration	30	400	3
Loading	Feed mixture	300	As in Table 2
Wash	30	150	2
Gradient	30–100	200	As in Table 2
Strip	100	150	2

**Table 2:** Loading and gradient lengths of linear gradient elution experiments. CV: column volume.

Exp no.	Label	Load (g/L_resin_)	Percentage of benchmark	Gradient length (CV)	Percentage of benchmark	Purpose
1	Load15_GL6p5	15	100%	6.5	100%	Training
2	Load7p5_GL6p5	7.5	50%	6.5	100%	Training
3	Load15_GL8p5	15	100%	8.5	130%	Training
4	Load15_GL4p6	15	100%	4.6	70%	Testing
5	Load22p5_GL6p5	22.5	150%	6.5	100%	Testing
6	Load22p5_GL8p5	22.5	150%	8.5	130%	Testing

All fractions collected from the elution experiments were analyzed by reversed‐phase high‐performance liquid chromatography. The analytical runs were performed using a YMC Triart C18 column (100 × 2 mm, particle size = 1.9 μm, porosity = 12 nm) on an Agilent 1200 HPLC system, with detection at 300 nm using a diode‐array detector. The temperature and flow rate were constant to 50°C and 0.2 mL/min, respectively. The equilibration buffer was a 100 mM hexafluoro isopropanol + 4 mM triethylamine solution. The elution buffer was pure methanol. The buffers were filtered through 0.2 μm PVDF membranes and degassed before usage. The system was equilibrated at 5% elution buffer for 1 min, before a gradient to 10% elution buffer in 2 min, from 10% to 15% in 22 min and to 90% in additional 2 min. Finally, re‐equilibration at 5% elution buffer was conducted for 15 min.

Impurities eluted within 8 min after the gradient start were considered negligible, as they did not co‐elute with the main product (P) during preparative experiments. Impurities co‐eluting in the front of P during preparative runs were grouped as weakly adsorbed impurities (W), and were separated via HPLC between 8 and 30.2 min. The product P eluted at 30.2 min. Species eluting after the product P were identified as strongly adsorbed impurities (S). More details about data collection and analytical method can be found in the single column methods of our previous work (Fioretti et al. 2024).

### 3.2 Model Implementation in Pytorch

The three operators (F, NN, and SOL) were implemented using PyTorch‐supported tensor formats, with their corresponding gradients computed through PyTorch AD framework. All computations were executed on an Intel i9‐13900K CPU utilizing single‐precision floating‐point arithmetic, without GPU acceleration.

The operator NN involved linear layers from PyTorch as FCNNs, with seven input nodes, three output nodes, and $n_{L}$ hidden layers (each with $n_{l}$ nodes). Weight initialization for the linear layers was performed using the Kaiming method (He et al. 2015). ReLU served as the activation function between linear layers, while Softplus was utilized as the activation function for the output layer. The MSE function, Equation (34), was adopted as the loss function. For the operator SOL, the linear system in each iteration of the BDF method was solved using the LU decomposition method provided by PyTorch.

### 3.3 Network Initialization Based on Mechanistic Models

The NNs were initialized using a mechanistic model. This was preliminarily calibrated through the standardized approach proposed by Chen et al. (2024), including a parameter‐by‐parameter method followed by an inverse method (Chen et al. 2022, 2023; Yang et al. 2024; Yang et al. 2024). It is worth underlying that this training set used to calibrate the mechanistic model was identical to the one used afterwards to train DP‐SOL. The loss function, normalization methods, data set partitioning for mechanistic modeling were consistent with those used in DP‐SOL. Once the network initialization was completed, the mechanistic model parameters remained unchanged throughout the NN training process.

### 3.4 Hyperparameter Selection

Before formal NN training, we utilized grid search to select the relevant hyperparameters. The NN underwent 20 epochs on the training set during each iteration. The random seed for layer initialization was consistent across iterations.

### 3.5 Learning Procedure

The learning procedure included: (1) partitioning of the experimental datasets into training and testing sets; (2) independent obtainment of a calibrated mechanistic model from the training set for initializing NNs; (3) selection of the relevant hyperparameters; (4) training of DP‐SOL on the training set for 2000 iterations under the selected hyperparameters; (5) evaluation of the extrapolability of the trained DP‐SOL to the testing set.

## 4 Results

### 4.1 Data Preparation and Partitioning

A conscious data set partitioning in training and test sets can ensure the model performs well on unseen data. To prepare the data set, we followed a one‐factor‐at‐a‐time approach starting from the experimental conditions reported in Table 1 with a gradient length of 6.5 CV and a loading of 15 g/L_resin_. A total of six experiments, varying the gradient length and loading by ±30% and ±50%, respectively, were divided into a training set (experiments No. 1, 2, and 3 in Table 2) and a testing set (experiments No. 4, 5, and 6 in Table 2) in a 1:1 ratio. From these runs, we collected fractions during the gradient and analyzed them via HPLC to detect and quantify the different species. Among the different components identified, we lumped together those species showing similar behavior. In particular, the impurities eluting earlier then the product were grouped in the pseudo‐component W. These are mainly represented by shortmers, and in particular by the *n‐1* oligonucleotide. On the other side, the impurities eluting later than the product were grouped in the pseudo‐component S. Therefore, the multicomponent system was reduced to a ternary mixture comprising W, P and S.

### 4.2 Neural Network Initialization Based on Mechanistic Model

NNs were initialized by a calibrated mechanistic model, which also serves as a benchmark for comparison with the hybrid model. The parameters used for the mechanistic model were derived according to the methods reported in Section 3.3 and are listed in Table 3. These parameters are physically meaningful. For example, ν increases moving from W to P and S, and in turn with their retention times.

**Table 3:** Mechanistic model parameters of weakly adsorbed impurities (W), main product (P), and strongly adsorbed impurities (S).

Component	ν	$k_{\text{eq}}$	σ	$k_{\text{kin}}$
W	6.08	7.38E‐5	29.17	2.89E‐7
P	7.70	7.31E‐6	12.49	4.49E‐10
S	12.63	8.15E‐9	0	1.56E‐12

The simulated elution curves based on these model parameters are shown in Figure S1 in Supporting Information. Due to significant differences in the content of product and impurities in the crude mixture, a more convenient evaluation of the model performance was provided in Figure S2, where the concentration at the column outlet is normalized by the corresponding one in the feed. Through this model, the averaged losses were $L_{\text{train},\text{MM}}\approx 1.1\times 10^{-1}$ and $L_{\text{test},\text{MM}}\approx 2.6\times 10^{-1}$ for the training and test set, respectively. These values suggest that the mechanistic model can already provide a good representation of the RPC process for this oligonucleotide. Indeed, the retention time is accurately captured.

However, the peak shapes were not precisely modeled. Specifically, for the impurity S, the measured peak is tailing (Langmuirian adsorption) while the simulated one is fronting (anti‐Langmuirian adsorption), resulting in its $R^{2}$ being lower than those for the impurity W and the product P (especially for Figure S2D). According to our previous work (Chen et al. 2025), interpreting adsorption types based on peak symmetry (tailing vs. fronting) requires constant flow conditions. However, as shown by the flow rate profiles in Figures S1 and S2, the elution flow rate was not constant during the elution of the impurity S. Therefore, peak symmetry alone cannot be used to reliably determine the adsorption mechanism in this case.

Additionally, as the product P eluted, a significant variance is observed in the measured conductivity due to displacement effects (Chen et al. 2024; Fioretti et al. 2022). SDM can simulate this variance (Arkell et al. 2017; Arkell et al. 2018), but fails to be fully consistent with experiments.

### 4.3 Hyperparameter Selection

Starting from the results of the mechanistic model, we initialized DP‐SOL and investigated the effects of the hyperparameters (the number of hidden layers $n_{L}$, the number of nodes $n_{l}$, and the learning rate α) introduced by the operator NN.

For the operator F, also the order $n_{p}$ and the number of elements K have an effect on the accuracy and computational cost of the discretization (Breuer et al. 2023; Javeed et al. 2011; Meyer et al. 2020). However, the investigation of $n_{p}$ and K are beyond the scope of this study, and they were fixed to 3 and 7, respectively. For the operator SOL, the integration step size was fixed at 5‰ of the total simulation time.

For the operator NN, the search ranges of $n_{L}$ and $n_{l}$ were determined as follows: a NN with $n_{L}=2$ can approximate any continuous function, hence $n_{L}\in \left\{1,2,3\right\}$; the number of input and output nodes of the operator NN are 7 and 3, respectively, so $n_{l}$ was set from 3 (equal to the output node number) to 21 (three times the input node number). The activation function was not considered as a hyperparameter. We expected the sign of the input to the operator SOL to be controlled solely by the operator F. This requirement can be met by the constantly positive Softplus, which equals to ReLU for the sufficiently large input. Adam optimizer performs better for few‐shot learning compared to other optimizers and was not considered as a hyperparameter. PyTorch recommends $\alpha =10^{-4}$ for the Adam optimizer, hence $\alpha \in \left\{10^{-4},10^{-3},10^{-2},10^{-1}\right\}$.

**Figure 2:**
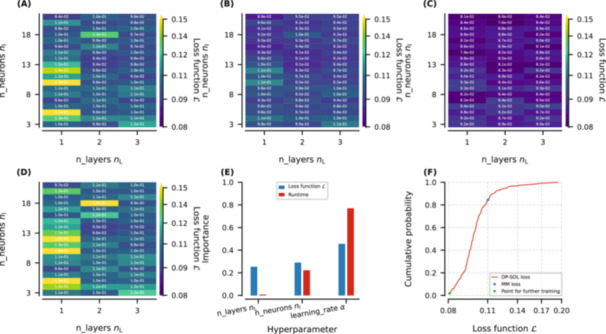
Hyperparameter search results: Contours of loss function between layer number and neuron number at learning rates of: (A) 10^−^^1^, (B) 10^−^^2^, (C) 10^−^^3^, and (D) 10^−4^. (E) Hyperparameter importance for loss function and runtime. (F) Cumulative probability of empirical distribution.

The hyperparameter search results in Figure 2A–D demonstrate the effects of NN structures ($n_{L}$ and $n_{l}$) on L at different α. The optimal α is $10^{-3}$ as identified in Figure 2C. A comparison of Figure 2A–D reveals that both excessively large or small α may cause a high L. In fact, a too large α may cause drastic fluctuations in L, making the training process unstable and convergence difficult to reach; while a too small α may result in very slow model convergence or in getting trapped in local optima.

Additionally, increasing $n_{L}$ does not bring much performance improvement, consistent with Ding et al. (2023) findings. The NN with 14 nodes and 2 layers shows nearly optimal performance across various α. According to Linoff and Berry (2011), NNs can achieve optimal performance when $n_{l}$ does not exceed twice the input node number (which is 7 in this study, making the upper limit 14).

The effects of these three hyperparameters are quantitatively evaluated in Figure 2E, exploring the importance and sensitivity of hyperparameters. This exploration was implemented through AD, which has been employed to explore the sensitivity of chromatographic model parameters (Püttmann et al. 2016; Püttmann et al. 2013). Similarly, it can extend to hyperparameters. Figure 2E indicates that the effects of $n_{L}$ and $n_{l}$ on both L and training time are relatively low, while α has a dominant effect.

Figure 2F shows the cumulative probability of the empirical distribution of these grid searches. The blue dot represents the calibration error of the mechanistic model, indicating that during the search process, approximately 85% of the hybrid model outperforms the mechanistic models after only 20 epochs. This demonstrates the significant improvement brought by DP‐SOL.

Considering NN performance, prevention of overfitting, computational effort, and training time, we considered $n_{L}=$ 2, $n_{l}=$ 14, and $\alpha =10^{-3}$ as a good starting point for further training. Its corresponding L is shown as a green dot in Figure 2F, where it is possible to observe that it was lower than the values obtained with 98% of other sets of hyperparameters. It is worth mentioning that this optimal set does not lie on the boundaries, hence the search ranges of hyperparameters were reasonable.

### 4.4 Model Training

**Figure 3:**
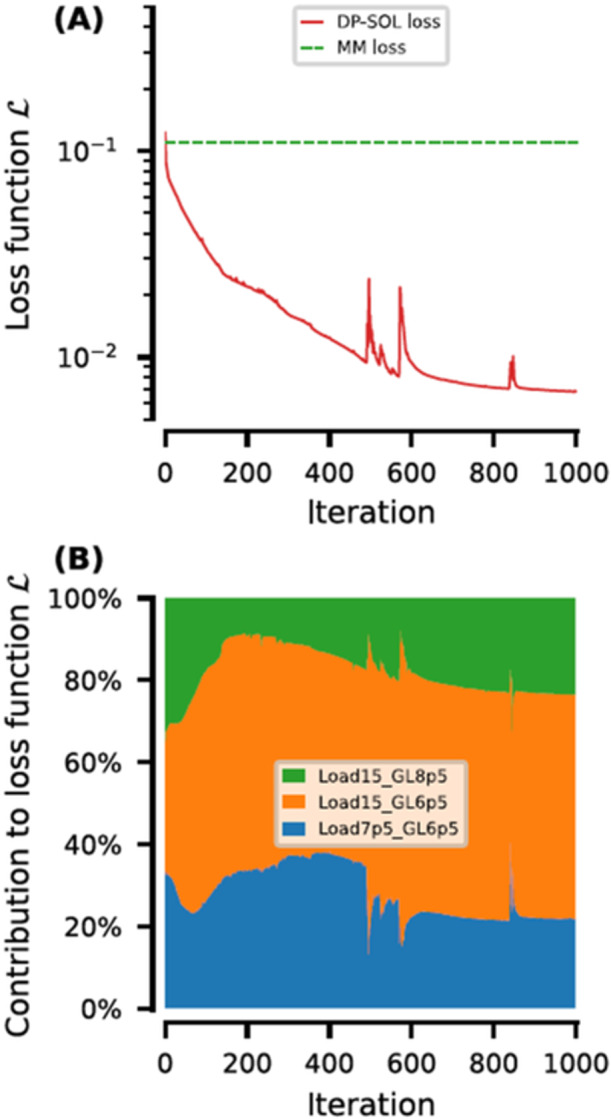
(A) Loss function from DP‐SOL and mechanistic model (MM) during consecutive iterations. (B) Contribution to the loss function from the three different experiments during model training.

Using the selected hyperparameters ($n_{L}=$ 2, $n_{l}=$ 14, and $\alpha =10^{-3}$), DP‐SOL was trained 2000 epochs (100 times the number of hyperparameter selections) with the evolution of $L_{\text{train},\text{DPSOL}}$ with the number of iterations shown in Figure 3A. The initial L after NN initialization is $1.1\times 10^{-1}$, which is close to $L_{\text{train},\text{MM}}$ obtained with the mechanistic model. This indicates that NNs have received good initialization and normalization, without significant fluctuations introduced by NNs. From the second epoch, $L_{\text{train},\text{DPSOL}}$ remains below $L_{\text{train},\text{MM}}$ and steadily decreases with increasing iterations until convergence. Training for 2000 epochs is reasonable, as the model reaches the optimal at the 988th epoch with $L_{\text{train},\text{DPSOL}}=6.8\times 10^{-3}$, which is significantly smaller than $L_{\text{train},\text{MM}}$.

Figure 3B illustrates the contributions of the three experiments used for training to the total $L_{\text{train},\text{DPSOL}}$. With increasing iterations, $L_{\text{train},\text{DPSOL}}$ is dominated by the experiment at a loading of 15 g/L_resin_ and a gradient length of 6.5 CV (orange shaded area). This is likely due to the relatively high error associated with the impurity S in this experiment.

**Figure 4:**
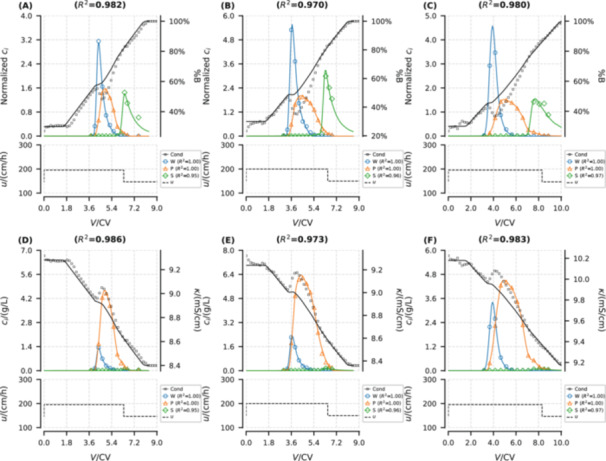
Trainig results of DP‐SOL hybrid models: normalized (A–C) and unnormalized (D–F) chromatograms from model simulations (solid lines) and experimental measurements (scatters) for weakly adsorbed impurities (W), main product (P), and strongly adsorbed impurities (S). (A) and (D): 7.5 g/L_resin_ loading and 6.5 CV gradient length. (B) and (E): 15 g/L_resin_ loading and 6.5 CV gradient length. (C) and (F): 15 g/L_resin_ loading and 8.5 CV gradient length. CV: column volume. Overall and individual $R^{2}$ are provided in the brackets in the titles and legend, respectively.

The elution curves simulated by the trained DP‐SOL are depicted in Figure 4. The normalized chromatograms (Figure 4A–C) show that DP‐SOL can accurately reproduce the experimental measurements in terms of both conductivity curves and elution curves of oligonucleotides during RPC processes (overall $R^{2}$ exceeds 0.97, and individual $R^{2}$ were also provided in brackets for each component in the legend).

Regarding the conductivity curves (black lines in Figure 4), the developed model exhibits similar variations with the measurements, in spite of failing to precisely replicate the magnitude.

Regarding the elution curves of oligonucleotides (colored lines in Figure 4), satisfactory fitting results were achieved in peak height, shape, and retention time, across the different loadings and gradient lengths. For the impurities W and S, despite their low concentrations, the model can remarkably simulate their elution curves. This ideal outcome is closely related to the normalization, including inputs of the operator NN, total outputs of DP‐SOL, and L. The normalization effectively scales features to similar ranges, mitigating the dominance of certain features in gradient updates and thus reducing the risk of gradient vanishing or exploding. In unnormalized chromatograms (Figure 4D–F), the overall $R^{2}$ almost equals the individual $R^{2}$ of the product P because its concentration is higher than those of impurities (W and S) by an order of magnitude. Conversely, in normalized chromatograms (Figure 4A–C), all peaks are scaled to have roughly the same peak areas, enhancing the reflection of overall fitting in $R^{2}$.

A comparison based on the same experimental data set, considering chromatograms normalized following identical approach, reveals that the hybrid model (Figure 4A–C) inherits the learning capability of NNs and can capture the adsorption behavior of the impurity S under varying velocities, despite the simplicity of both the column model and NN architecture, and the hybridization being applied solely to the adsorption model. This was not possible instead with the mechanistic model (Figure S2 in Supporting Information), showing large inaccuracy for this pseudo‐component.

### 4.5 Model Testing

**Figure 5:**
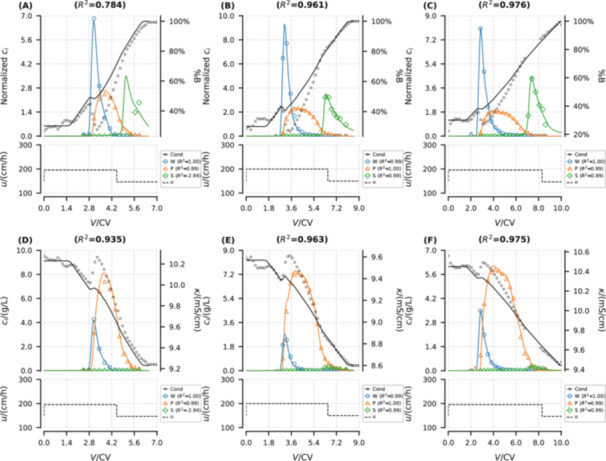
Testing results of DP‐SOL hybrid model: normalized (A–C) and unnormalized (D–F) chromatograms of model simulations (solid lines) and measurements (scatters) for weakly adsorbed impurities (W), main product (P), and strongly adsorbed impurities (S). (A) and (D): 15 g/L_resin_ loading and 4.6 CV gradient length. (B) and (E): 22.5 g/L_resin_ loading and 6.5 CV gradient length. (C) and (F): 22.5 g/L_resin_ loading and 8.5 CV gradient length. CV: column volume. Overall and individual $R^{2}$ are provided in the brackets in the titles and legend, respectively.

The trained DP‐SOL was then applied to the simulation of the elution conditions selected for testing (experiments No. 4, 5 and 6 in Table 2). Figure 5 reveals that the trained DP‐SOL can excellently predict the elution curves at gradient lengths and loading conditions different from those considered for model training, despite some oscillations in certain curves (peak position of product P).

During the hybrid model data set preparation, a one‐factor‐at‐a‐time design principle (i.e., varying either elution gradient length or loading) was adopted to ensure that the model could capture the individual effects of these variables. As summarized in Table 3, different velocities were applied across the various chromatographic steps. The effect of changing velocity was accounted for into the hybrid model through the convection and dispersion terms in Equation (20), where $D_{\text{app}}$ was modeled as flow‐dependent in accordance to Equation (35).

However, $R^{2}$ in Figure 5A (15 g/L_resin_ loading and 4.6 CV gradient length) is only 0.784, primarily due to the poor predictivity for impurity S, as it can be deduced from its individual $R^{2}$. The impurity S was experimentally detected in only two fractions, because analytical results showed that the impurity S accounted for only 1.2% of the crude mixture, and the low concentration may limit the accuracy of quantification. This can be observed in the unnormalized chromatogram (Figure 5D), where the elution curve of impurity S approaches a horizontal line. The loss in model performance due to anomalous datasets does not justify poor extrapolation of DP‐SOL. This experiment was used as a testing rather than for model training, effectively minimizing the impact of analytical accuracy on model development. Despite having only two data points, the hybrid model was still able to predict the peak shape reasonably well.

## 5 Discussion

In this study, the training set comprises only three linear gradient elution experiments. In contrast to purely data‐driven models relying on extensive data set, this few‐shot learning strategy of DP‐SOL offers convenience to users in data preparation. This is particularly important in the biopharmaceutical field, characterized by limited data availability and high experimental costs. The cost for preparing the data set of DP‐SOL is comparable to traditional mechanistic models, enabling users to seamlessly transition from conventional mechanistic models to hybrid ones without incurring additional experimental costs. Furthermore, it allows users to compare the performance of different models on the same data set.

Unlike most data‐driven models (interpolative prediction), DP‐SOL is assessed through extrapolative prediction. The model is trained using gradient lengths ranging from 6.5 to 8.5 CV and loadings from 7.5 to 15.0 g/L_resin_, while predictions are made at a gradient length of 4.6 CV and a loading of 22.5 g/L_resin_. Therefore, we ensure that there is no overlap between the training and testing sets, making them mutually independent.

In the model training, the gradient‐based optimization strategy using AD and backpropagation led to efficient NN training. This method can be equally effective in higher‐dimensional parameter spaces and deeper NNs, where the least squares method is inadequate. In the model testing, DP‐SOL exhibited a significant performance improvement compared to the purely mechanistic model. We attributed the excellent extrapolability of DP‐SOL to its two components.

On one hand, contribution stems from the mechanistic (white‐box) model, for example, the operator F. SDM performs well in predicting different gradient lengths in the presence of organic solvents, an advantage that has been inherited by DP‐SOL. However, recent research indicates that SDM fails in predicting high loadings (Koch et al. 2022; Seelinger et al. 2022; Seelinger et al. 2023; Seelinger et al. 2022), as corroborated in Figure S2E,F (high loadings), which exhibits larger testing errors compared to other experiments at low loadings.

On the other hand, the contribution of the data‐driven (black‐box) model, for example, the operator NN. The introduction of NNs enables DP‐SOL to interpret unknown mechanisms (Park et al. 2023; Schiemer et al. 2023), such as elution behavior at high loadings, despite employing a very simple column model and shallow NNs, while only considering the contribution of oligonucleotides to the stationary phase. When Ding et al. (2023) utilized NNs to develop a hybrid model for hydrophobic interaction chromatography, the authors considered separate NNs for mass transfer resistance and adsorption isotherms, coupling them to obtain the final output. In comparison, DP‐SOL serves as a general modeling paradigm at a solver level. In fact, it eliminates the need for multiple NNs or careful investigation of integration between NNs and PDEs. Therefore, DP‐SOL is simpler and more user‐friendly.

In terms of interpretability, DP‐SOL retains the physical transparency of key transport phenomena, such as mass transfer, dispersion, and convection, inherited from the mechanistic model. Only the adsorption behavior is learned via the NN operator, enabling the model to generalize beyond its training conditions while maintaining partial interpretability. This structure allows users to trace how input conditions propagate through physical mechanisms to yield model outputs, which is particularly valuable in biopharmaceutical process development where understanding of critical process parameters is essential. From a Quality by Design perspective, DP‐SOL might support process optimization, risk assessment, robustness evaluation, and regulatory acceptance more effectively than purely data‐driven models.

Overall, the hybridization of DP‐SOL leverages the advantages of mechanistic and data‐driven models. DP‐SOL possesses the capability of data‐driven models to interpret unknown mechanisms, which supplements known mechanistic models. A mechanistic model with poor performance can be transformed into a perfect hybrid model using DP‐SOL. Actually, DP‐SOL falls within the realm of operator learning. By counting the number of three operators, we observed that for each time step in the loop of DP‐SOL, there are 12 operators contributed by NNs for learning and 1920 operators determined by mechanistic models. We calculated the degree of hybridization (proposed by Narayanan et al. [2022]) in this study to be approximately 0.6%. This implies that DP‐SOL leans more towards mechanistic modeling rather than data‐driven modeling. This elucidates why DP‐SOL exhibits characteristics such as few‐shot learning, interpretability, and excellent extrapolability, typical of mechanistic models.

However, DP‐SOL also inherits the drawbacks of both mechanistic and data‐driven models, primarily evident in its implementation. All issues that need to be considered in the implementation of mechanistic and data‐driven models must be reevaluated in DP‐SOL, including governing equations, discretization, solver selection for mechanistic models, determination of NN structure, and hyperparameter selection for data‐driven models. Nevertheless, this also implies that knowledge of numerical methods from known mechanistic models will not be discarded compared to purely data‐driven models. Another drawback lies in computational costs. The computational time of DP‐SOL approaches the sum of computational times for mechanistic and data‐driven models. Benefitting from efficient numerical methods like DG‐FEM and advancements in distributed learning and computing power, DP‐SOL will unleash more applications in the future.

As a novel and versatile hybrid modeling paradigm, DP‐SOL has the potential to significantly impact modeling approaches in the downstream processing field and the broader biopharmaceutical field. By altering the governing equations, discretization forms, solvers, and NN structures, DP‐SOL can be applied to various processes (Andersson et al. 2023; Ding et al. 2022; Gomis‐Fons et al. 2024; Tiwari et al. 2023). For example, it can be used in the process design and optimization of multi‐column counter‐current solvent gradient purification (MCSGP) of oligonucleotides. The gradient information in DP‐SOL can conveniently establish the mapping between optimization objectives and decision variables, thereby solving MCSGP optimization problems more efficiently.

## 6 Conclusion

This study introduced a hybrid modeling framework named DP‐SOL, which integrates traditional mechanistic models with NNs. The framework utilizes differentiable physical operators and their gradients, and was applied to describe the RPC purification of an oligonucleotide. We first collected a small data set for few‐shot learning by performing six linear gradient elution experiments at different resin loadings and gradient slopes, with three experiments each for training and testing in a 1:1 ratio. The NN was initialized using a calibrated mechanistic model. Hyperparameters were determined through a grid search, resulting in a NN with 2 hidden layers and 14 nodes. Compared to a purely mechanistic model, the DP‐SOL hybrid model showed significant performance improvement, invariably achieving a higher $R^{2}$ on both training and testing sets, with $R^{2}$ > 0.97 for the former. We attributed the robust predictive capability of DP‐SOL to the combination of mechanistic models and NNs at the solver level. As a novel and versatile hybrid modeling paradigm, DP‐SOL has the potential to significantly impact the chromatographic purification of biologics, establishing digital tools as key resources for reduced process development times and costs.

## Author Contributions

**Yu‐Cheng Chen:** methodology, software, investigation, visualization, writing – original draft. **Ismaele Fioretti:** investigation, validation, data curation. **Dong‐Qiang Lin:** supervision, funding acquisition, writing – review and editing. **Mattia Sponchioni:** project administration, supervision, funding acquisition, writing – review and editing.

## Conflicts of Interest

The authors declare no conflicts of interest.

## Supporting information

DP‐SOL_for_RPC_B_SI.

## Data Availability

The data that support the findings of this study are available from the corresponding author upon reasonable request.
