# Supplementary material for: Hybrid Modeling of the Reversed‐Phase Chromatographic Purification of an Oligonucleotide: Few‐Shot Learning From Differentiable Physics Solver‐in‐the‐Loop
Source: Biotechnol Bioeng. 2025 May 9;122(8):2179–92. doi: 10.1002/bit.29018 (PMC12235242; doi:10.1002/bit.29018)
Supplement: Supplementary file 1 — DP‐SOL_for_RPC_B_SI. [file BIT-122-2179-s001.pdf]

Supporting Information

Hybrid modeling of the reversed-phase chromatographic purification of an oligonucleotide: Few-shot learning from differentiable physics solver-in-the-loop

Yu-Cheng Chen[a,b], Ismaele Fioretti[b], Dong-Qiang Lin[a], Mattia Sponchioni[b,*]

a. Key Laboratory of Biomass Chemical Engineering of Ministry of Education, Zhejiang Key Laboratory of Smart Biomaterials, College of Chemical and Biological Engineering, Zhejiang University, Hangzhou 310058, China
b. Department of Chemistry, Materials and Chemical Engineering "Giulio Natta", Politecnico di Milano, Via Mancinelli 7, 20131 Milano, Italy

***Corresponding author:**

Prof. Mattia Sponchioni

mattia.sponchioni@polimi.it

Politecnico di Milano

Dpt. of Chemistry, Materials and Chemical Engineering "Giulio Natta"

via Mancinelli, 7

20131 Milano, Italy

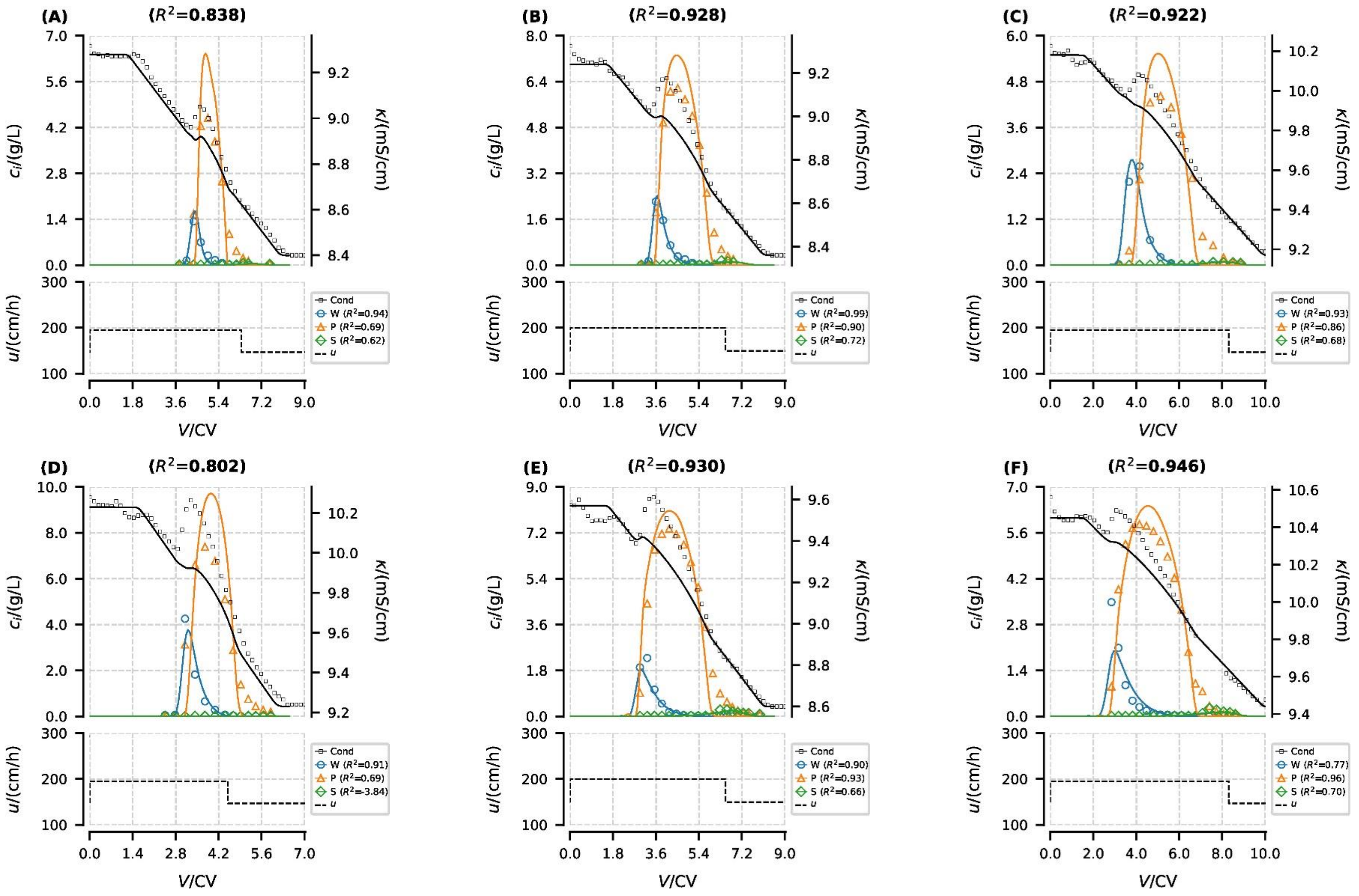

Fig S1. Unnormalized chromatograms obtained experimentally (scatters) and simulated by mechanistic model with the parameters shown in Table 3 (solid lines) for weakly adsorbed impurities (W), main product (P), and strongly adsorbed impurities (S). Training set: (A) 7.5 g/L$_{resin}$ loading and 6.5 CV gradient length, (B) 15 g/L$_{resin}$ loading and 6.5 CV gradient length, and (C) 15 g/L$_{resin}$ loading and 8.5 CV gradient length. Testing set: (D) 15 g/L$_{resin}$ loading and 4.6 CV gradient length, (E) 22.5 g/L$_{resin}$ loading and 6.5 CV gradient length, and (F) 22.5 g/L$_{resin}$ loading and 8.5 CV gradient length. CV: column volume. Overall and individual R^2 are provided in the brackets in the titles and legend, respectively.

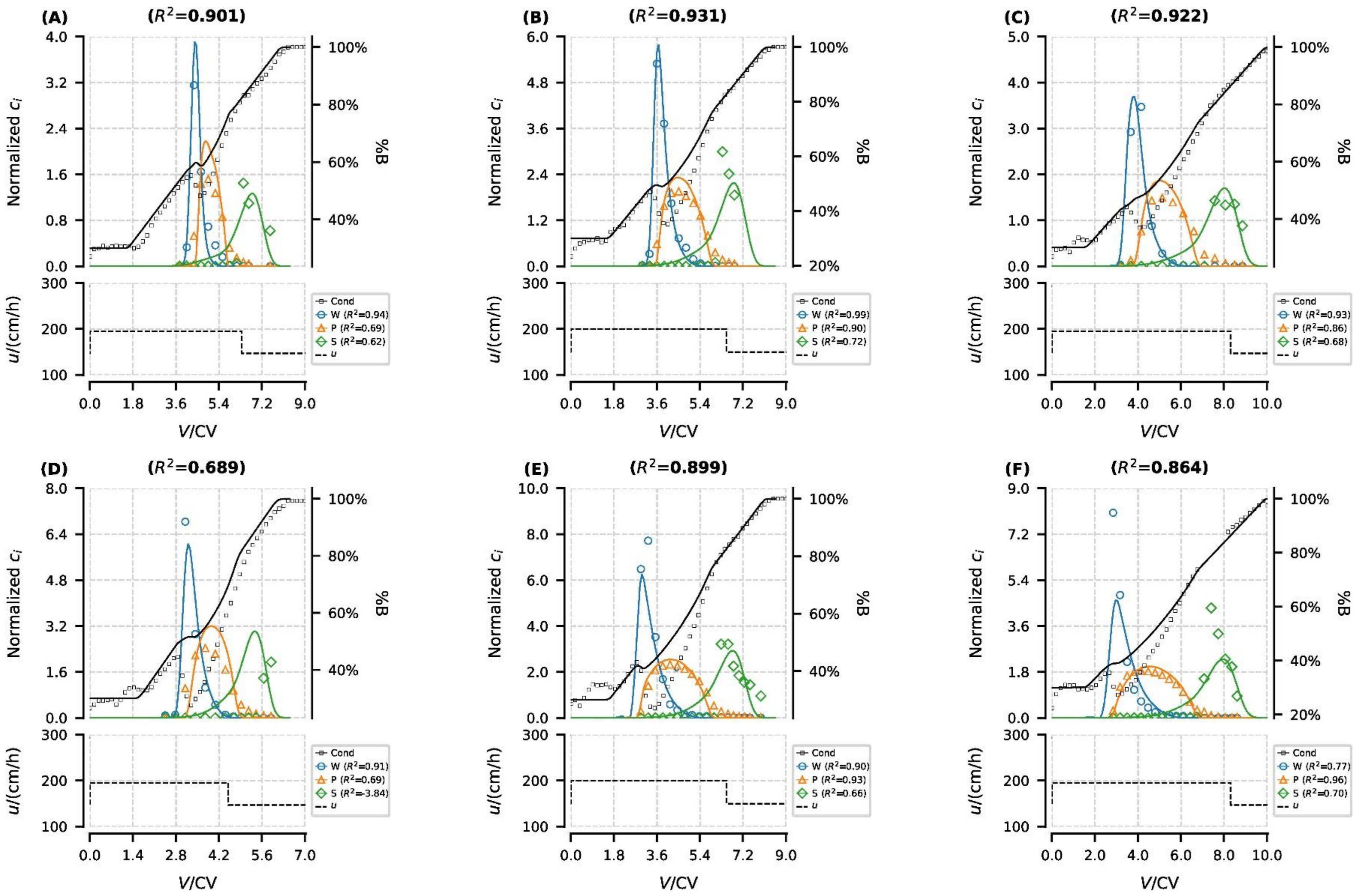

Fig S2. Normalized chromatograms obtained experimentally (scatters) and simulated by mechanistic model with the parameters shown in Table 3 (solid lines) for weakly adsorbed impurities (W), main product (P), and strongly adsorbed impurities (S). Training set: (A) 7.5 g/L$_{resin}$ loading and 6.5 CV gradient length, (B) 15 g/L$_{resin}$ loading and 6.5 CV gradient length, and (C) 15 g/L$_{resin}$ loading and 8.5 CV gradient length. Testing set: (D) 15 g/L$_{resin}$ loading and 4.6 CV gradient length, (E) 22.5 g/L$_{resin}$ loading and 6.5 CV gradient length, and (F) 22.5 g/L$_{resin}$ loading and 8.5 CV gradient length. CV: column volume. Overall and individual R^2 are provided in the brackets in the titles and legend, respectively.
